# A Conformation-Sensitive Monoclonal Antibody against the A2 Domain of von Willebrand Factor Reduces Its Proteolysis by ADAMTS13

**DOI:** 10.1371/journal.pone.0022157

**Published:** 2011-07-11

**Authors:** Jingyu Zhang, Zhenni Ma, Ningzheng Dong, Fang Liu, Jian Su, Yiming Zhao, Fei Shen, Anyou Wang, Changgeng Ruan

**Affiliations:** 1 Jiangsu Institute of Hematology, The First Affiliated Hospital of Soochow University, Suzhou, China; 2 Department of Hematology, Hebei Institute of Hematology, The Second Hospital of Hebei Medical University, Shijiazhuang, China; 3 Emory University School of Medicine, Atlanta, Georgia, United States of America; 4 Key Laboratory of Thrombosis and Hemostasis of Ministry of Health, The First Affiliated Hospital of Soochow University, Suzhou, China; Leiden University Medical Center, The Netherlands

## Abstract

The size of von Willebrand factor (VWF), controlled by ADAMTS13-dependent proteolysis, is associated with its hemostatic activity. Many factors regulate ADAMTS13-dependent VWF proteolysis through their interaction with VWF. These include coagulation factor VIII, platelet glycoprotein 1bα, and heparin sulfate, which accelerate the cleavage of VWF. Conversely, thrombospondin-1 decreases the rate of VWF proteolysis by ADAMTS13 by competing with ADAMTS13 for the A3 domain of VWF. To investigate whether murine monoclonal antibodies (mAbs) against human VWF affect the susceptibility of VWF to proteolysis by ADAMTS13 in vitro, eight mAbs to different domains of human VWF were used to evaluate the effects on VWF cleavage by ADAMTS13 under fluid shear stress and static/denaturing conditions. Additionally, the epitope of anti-VWF mAb (SZ34) was mapped using recombinant proteins in combination with enzyme-linked immunosorbent assay and Western blot analysis. The results indicate that mAb SZ34 inhibited proteolytic cleavage of VWF by ADAMTS13 in a concentration-dependent manner under fluid shear stress, but not under static/denaturing conditions. The binding epitope of SZ34 mAb is located between A1555 and G1595 in the central A2 domain of VWF. These data show that an anti-VWF mAb against the VWF-A2 domain (A1555-G1595) reduces the proteolytic cleavage of VWF by ADAMTS13 under shear stress, suggesting the role of this region in interaction with ADAMTS13.

## Introduction

Plasma von Willebrand factor (VWF) is a large multimeric glycoprotein that interacts with platelet surface receptors and is crucial for normal hemostasis. The adhesive activity of VWF is positively correlated with the size of the multimers in plasma [1], [2]. VWF multimer size is regulated by metalloproteinase ADAMTS13, which cleaves the central A2 domain of VWF at the Tyr1605-Met1606 bond [3]–[4]. The importance of VWF proteolysis by ADAMTS13 is demonstrated in two syndromes, i.e., thrombotic thrombocytopenic purpura and von Willebrand disease type 2A. The former is associated with the deficiency of plasma ADAMTS13 activity, either due to congenital mutations or acquired autoantibodies [5]–[7]. The latter is mostly caused by mutations in the A2 domain of VWF that lead to the increased proteolysis of VWF multimers by ADAMTS13 [8], [9].

Many factors modulate the proteolysis of VWF by ADAMTS13. These ligands that bind the A1 domain of VWF such as platelet glycoprotein Ibα, heparin and ristocetin promote VWF proteolysis by ADAMTS13 [10]. In addition, platelets also significantly enhance the cleavage of VWF multimers by ADAMTS13 under fluid shear stress [11], [12]. On the contrary, thrombospondin-1, an extracellular matrix adhesion protein, may compete with ADAMTS13 for binding to the A3 domain of VWF, which reduces the rate of VWF proteolysis by ADAMTS13 [13], [14].

In this study, we investigated the effects of eight murine monoclonal antibodies (mAbs) against various domains of VWF on its proteolysis by ADAMTS13 under physiologically relevant conditions. Among those, mAb SZ34 dramatically decreased the susceptibility of VWF to ADAMTS13 under shear stress, but not under static/denaturing conditions. The epitope of SZ34 was mapped to the amino acid residues between A1555 and G1595 in the central A2 domain of VWF. Our findings highlight the critical role of this region for ADAMTS13-mediated proteolysis under shear stress.

## Results

### Anti-VWF mAb SZ34 decreases the susceptibility of VWF to proteolysis by ADAMTS13 under shear stress

**Figure 1:**
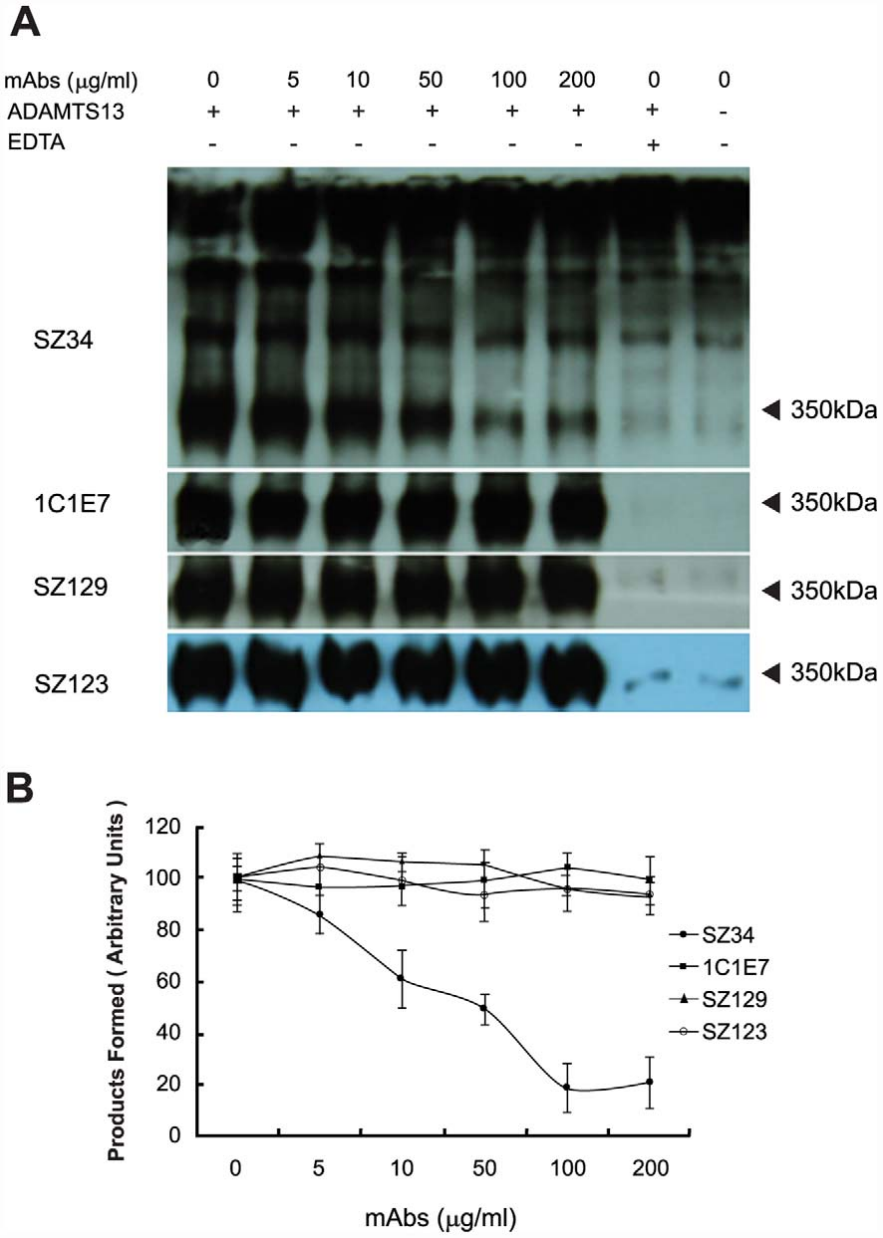
SZ34 inhibits the proteolytic cleavage of pVWF by rADAMTS13 under shear stress. (A) Purified pVWF (150 nM) was pre-incubated with SZ34 (0–200 µg/ml) for 30 min at 37°C, and then incubated with 50 nM rADAMTS13. After 5 min of vortexing at 2,500 rpm on a mini vortexer, the 350 kDa cleavage products were visualized by 5% SDS-PAGE under non-reducing conditions and Western blot analysis. 1C1E7 (an anti-VWF D'D3 mAb), SZ129 (an anti-VWF A1 mAb) and SZ123 (an anti-VWF A3 mAb) were used as negative controls. (B) Changes in the cleavage products detected relative to that observed in the absence of mAbs were determined under shear stress by densitometry. The extent of cleavage was analyzed by detection of the intensity of the 350 kDa cleavage products. Results represent the mean ± standard deviation of four independent experiments.

In this study, we used rADAMTS13 and pVWF as the sources of enzyme and substrate to determine the effect of SZ34 on VWF proteolysis under shear stress. The cleavage product was determined by SDS-PAGE under non-reducing conditions followed by Western blot. The results showed that the 350 kDa cleavage product of VWF under shear stress was reduced in the presence of SZ34 in a concentration-dependent manner (Figure 1). The half maximal (50%) inhibitory concentration (IC_50_) of the SZ34 was estimated to be approximately 50 µg/ml (Figure 1). In contrast, other anti-VWF mAbs including 1C1E7 (an anti-VWF D'D3 mAb), SZ129 (an anti-VWF A1 mAb), SZ123 (an anti-VWF A3 mAb) and so on had no effects on the proteolytic cleavage of VWF by ADAMTS13 (Figure 1 and Table 1). The control experiments established the lack of detectable proteolysis in the absence of ADAMTS13 or in the addition of 20 mM EDTA to reaction mixtures (Figure 1).

**Table 1:** Summary of 8 mAbs to VWF and their effects on VWF proteolysis by ADAMTS13.

mAbs	Epitope	Effects on VWF proteolysis by ADAMTS13	
		under shear stress	under static/denaturing conditions
1C1E7^25^	VWF-D'D3	no effect	no effect
75H4B12^25^	VWF-D'D3	no effect	no effect
SZ129^26^	VWF-A1	no effect	no effect
SZ130^26^	VWF-A1	no effect	no effect
SZ29^28^	VWF-A2	no effect	no effect
SZ34^27,28^	VWF-A2	inhibition	no effect
SZ123^27^	VWF-A3	no effect	no effect
SZ125^27^	VWF-A3	no effect	no effect

**Figure 2:**
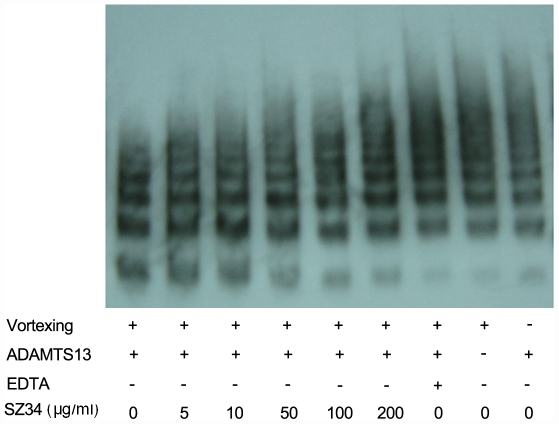
SZ34 inhibits the proteolysis of VWF multimers by ADAMTS13 under shear stress. Purified pVWF (150 nM) was pre-incubated with SZ34 (0–200 µg/ml) for 30 min at 37°C, and then incubated with 50 nM rADAMTS13. After 5 min of vortexing at 2,500 rpm on a mini vortexer, VWF multimers were separated by 1.5% agarose gel electrophoresis and immunologic analysis. A representative image of 4 independent experiments is shown.

The proteolysis of VWF by ADAMTS13 in the absence and presence of anti-VWF mAbs was also determined by agarose gel electrophoresis visualizing VWF multimers. We showed that the decreased amount of the high and intermediate molecular weight multimers were dramatically reduced by mAb SZ34 in a concentration-dependent manner under shear stress (Figure 2), further confirming the role of SZ34 in decreasing the susceptibility of VWF to proteolytic cleavage by ADAMTS13 under physiologically relevant conditions.

### SZ34 had no effects on VWF proteolysis by ADAMTS13 under denaturing/static condition

**Figure 3:**
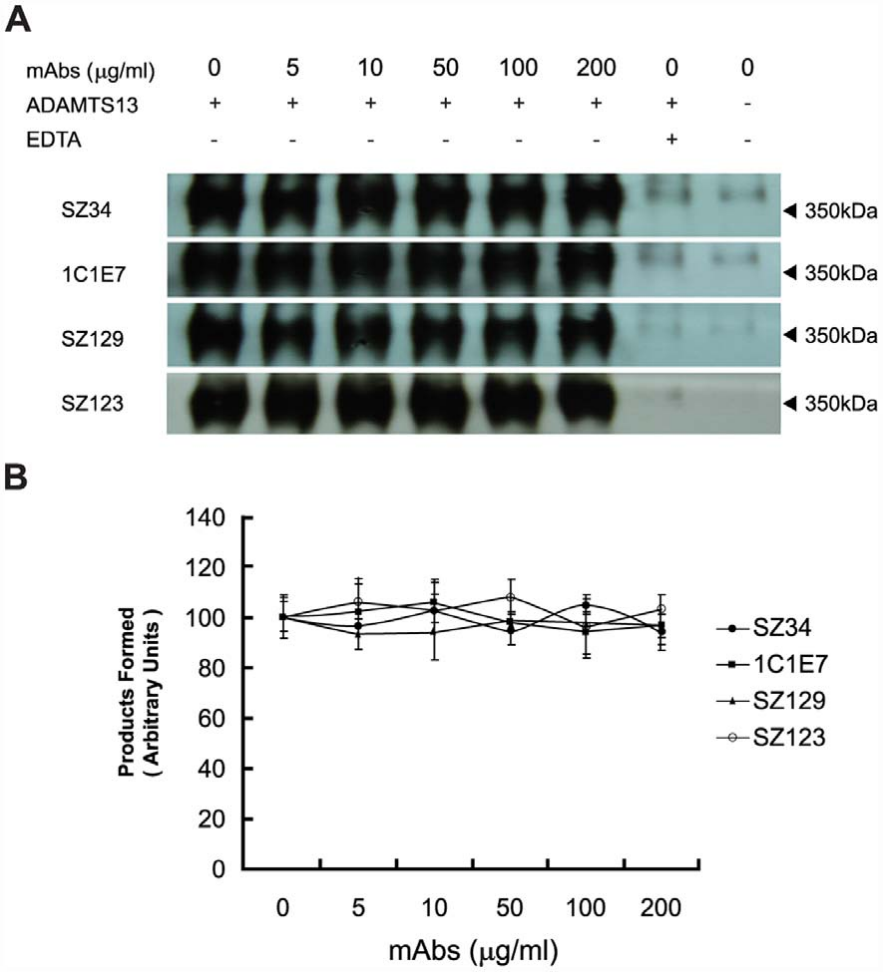
SZ34 has no effects on the proteolysis of denatured pVWF by rADAMTS13 under static conditions. (A) Purified pVWF (150 nM) pretreated with guanidine-HCl was incubated with SZ34 (0–200 µg/ml), and then incubated with 25 nM rADAMTS13. After 1.5 h, the reaction was quenched by adding 20 mM EDTA. The 350 kDa cleavage products were analyzed by Western blot as above. 1C1E7, SZ129 and SZ123 were used as controls. (B) Changes in the cleavage product detected relative to that observed in the absence of SZ34 were determined under denatured conditions by densitometry. The extent of cleavage was analyzed by detection of the intensity of the 350 kDa cleavage products. Results represent the mean ± standard deviation of four independent experiments.

When pVWF was pre-denatured with guanidine-HCl before incubation with SZ34 and rADAMTS13, no obvious difference in the 350 kDa cleavage products was detected (Figure 3 and Table 1). Thus, SZ34 had no effect on the digestion of unfolded VWF by ADAMTS13. In the meantime, anti-VWF mAbs including 1C1E7, SZ129, SZ123, etc, exhibited no effects on ADAMTS13-mediated proteolysis of the unfolded VWF under static conditions either (Figure 3 and Table 1).

**Figure 4:**
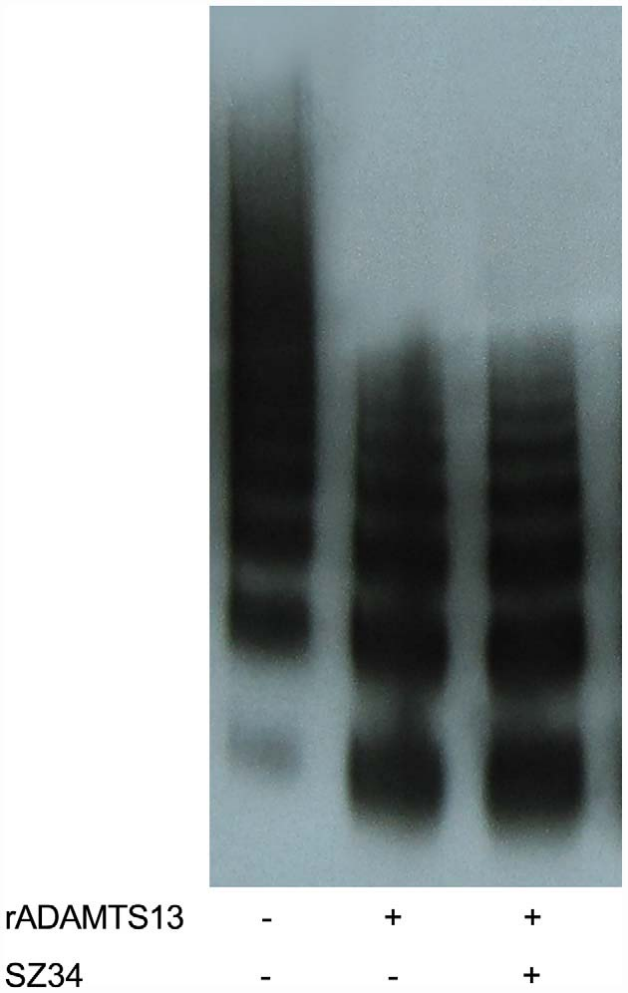
SZ34 had no effect on proteolysis of VWF-R1597W mutant by ADAMTS13. Recombinant VWF-R1597W (150 nM) was incubated with 0 or 100 µg/ml SZ34 at 37°C for 30 min and then for 18 h with 25 nM rADAMTS13 at 37°C. VWF multimers were separated by 1.5% agarose gel electrophoresis and immunologic analysis. A representative image of 4 independent experiments is shown.

To exclude the influence of denaturing condition on interaction between VWF and antibodies, we constructed and expressed VWF-R1597W mutant and investigated whether SZ34 inhibited its proteolysis by ADAMTS13. R1597W is the most frequent mutation in von Willebrand disease type 2A and the mutation site is adjacent to Tyr1605-Met1606 bond. This mutant VWF can be cleaved by ADAMTS13 under static condition and in the absence of denaturants such as urea and guanidine [8]. The results showed that with or without SZ34 R1597W VWF multimers were equally effectively cleaved by ADAMTS13 under static conditions (Figure 4), which suggested that SZ34 had no effects on the proteolysis of this VWF mutant by ADAMTS13. Preliminary experiments have shown that SZ34 binds to VWF- R1597W multimers by an ELISA method and the dissociation constants (Kd) is 0.67±0.11 nM.

### Epitope mapping of anti-VWF mAb SZ34

**Figure 5:**
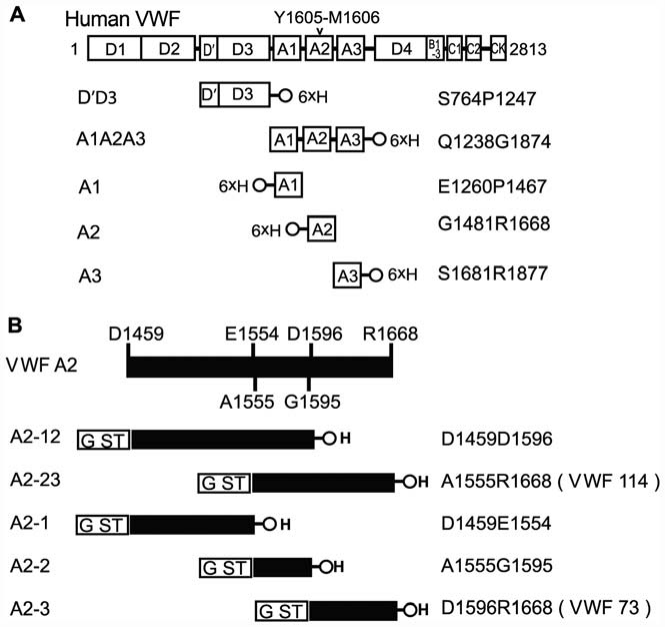
Schematic domain structure of full-length VWF and recombinant VWF fragments. The domain structure of human preproVWF is shown above the structures of recombinant VWF fragments designed in this study. ADAMTS-13 cleaves the Y1605-M1606 peptidyl bond in the A2 domain (D1459-L1668). Five different recombinant VWF domains with His-tags (Figure 5A) and 5 different recombinant proteins derived from the VWF A2 domain flanked with GST- and His-tags (Figure 5B).

To determine the binding epitope of SZ-34, we prepared a series of recombinant VWF fragments, including A1A2A3, A1, A2, A3 and D'D3 (Figure 5A), and five GST fusion VWF-A2 fragments, i.e. A2-1, A2-2, A2-3 (VWF73), A2-12 and A2-23 (VWF114) (Figure 5B). We measured the binding capacities (dissociation constants, Kd) of SZ34 to native and guanidine-denatured VWF and various VWF fragments by ELISA method. SZ34 bound to native and pre-denatured full-length VWF, A1A2A3, and A2, but only native A2-12 and A2-23 (Table 2). Furthermore, the binding of SZ34 to the pre-denatured VWF and VWF fragments was much weaker than the native counterparts (Table 2). These data suggest that the epitope of SZ34 is conformationally sensitive. The binding site was mapped to the central A2 domain of VWF. Because SZ34 bound to both native A2-12 and native A2-23, the epitope of SZ34 appeared to be located within the A2-2 (A1555-G1595 region).

**Table 2:** Kinetics of SZ34 and SZ29 interactions with native or denatured pVWF and various recombinant VWF fragments.

Various recombinant VWF fragments or pVWF	Kd (nM)	
	SZ34	SZ29
Native pVWF	4.7 (±0.6)×10^−2^	2.9 (±0.4)×10^−2^
Denatured pVWF	12.3±3.2	16.4±5.4
Native/denatured D'D3 (S764P1247)	>10^3^	>10^3^
Native A1A2A3 (Q1238G1874)	0.98±0.11	0.51±0.03
Denatured A1A2A3	53.7±6.9	46.3±4.1
Native/denatured A1 (E1260P1467)	>10^3^	>10^3^
Native A2 (G1481R1668)	5.2±0.8	3.3±0.5
Denatured A2	85.4±2.2	75.8±5.9
Native/denatured A3 (S1681R1877)	>10^3^	>10^3^
Native A2-12 (D1459D1596)	38.4±5.9	62.4±8.3
Denatured A2-12	>10^3^	>10^3^
Native A2-23 (A15555R1668)	26.1±8.8	>10^3^
Denatured A2-23	>10^3^	>10^3^
Native/denatured A2-1(D1459E1554)	>10^3^	>10^3^
Native/denatured A2-2 (A1555G1595)	>10^3^	>10^3^
Native/denatured A2-3 (D1596R1668)	>10^3^	>10^3^
BSA	>10^3^	>10^3^

Data represent mean ± SD.

**Figure 6:**
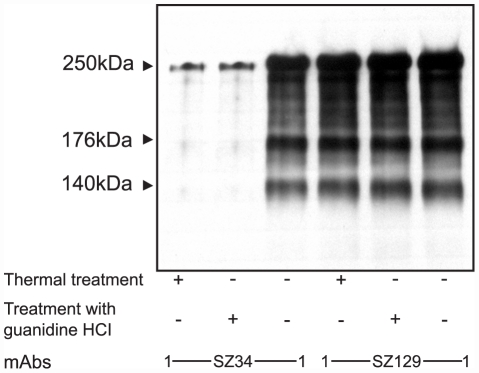
Comparison of the binding activity of SZ34 to native and denatured pVWF using Western blot in combination with ELISA based on polystyrene microspheres. SZ34 (20 µg/ml) was coated on polystyrene microspheres, then incubated with native pVWF or denatured pVWF (100 nM). The denatured pVWF was obtained by thermal treatment (20 min at 80°C) or treatment with 1.5 M guanidine-HCl (2 h at 37°C) of native pVWF. Bound VWF was separated on 6% SDS-PAGE in reducing conditions, followed by Western blotting with SZ34. SZ129 (anti-VWF A1) was a control as a mAb with a linear epitope. The figure is representative of four separate experiments.

To further to testify that SZ34 is a conformational mAb against VWF, a western blot in combination with ELISA based on polystyrene microspheres was performed to compare the binding activities of SZ34 to native pVWF and denatured pVWF. Compared with native pVWF, the binding activity of SZ34 to heated pVWF or pVWF treated with 1.5 M guanidine-HCl was significantly reduced (Figure 6), further confirming that SZ34 is a conformation-sensitive mAb to VWF.

## Discussion

We found that SZ34, a mAb against the A2 domain of human VWF, reduces the susceptibility of VWF to proteolysis by ADAMTS13 under fluid shear stress. However, this effect was not observed under static and chemically denaturing conditions. Seven other mAbs against VWF including SZ129 and SZ130 (two mAbs against VWF A1), SZ29 (another mAb against VWF A2), SZ123 and SZ125 (two mAbs against VWF A3) and 1C1E7 and 75H4B12 (two mAbs against VWF D'D3) exhibited no effects on ADAMTS13-mediated VWF proteolysis under either shear stress or denaturing conditions. The epitope mapping shows that the epitope of SZ-34 is located within the A1555-G1595 region of the A2 domain of native VWF, suggesting that this region may directly interact with ADAMTS13. Furthermore, the mAb SZ-34 is a conformation-sensitive mAb because it preferentially binds to native VWF.

Only two reports have been published showing the inhibitory effects of VWF antibodies on the proteolysis of VWF by ADAMTS13, but the mechanism of inhibition by SZ34 may be different from that of the two other mAbs VP-1 and RU8 reported by Tsai et al [15] and Zanardeli et al [16], respectively. The mAb VP-1, which was directly against residues 1591–1605 (inside VWF A2 domain) of VWF polypeptide, inhibited the susceptibility of the recombinant type 2A VWF mutants R1606W and R1606Q to proteolytic cleavage by ADAMTS13 under the static condition. But they did not examine whether VP-1 inhibited native pVWF or wild-type recombinant VWF proteolysis by ADAMTS13 [15]. Because VP-1 was raised against the synthetic VWF peptide (residues 1591–1605) and reacted poorly with the intact subunit (250 kDa fragment) of VWF in reduced SDS-PAGE followed by immunoblotting [3], [15], we speculated that VP-1 could not bind to native VWF and could not affect its proteolysis by ADAMTS13 without denaturization or under shear stress. In addition, Zanardelli et al [16] reported the inhibition of VWF proteolysis by ADAMTS13 under shear by using RU8, a mAb directed against the VWF D4 domain. They suggested that a binding site in the C-terminal region (D4CK) of VWF is constitutively exposed and participates at the initial step of a multi-step interaction, ultimately leading to proteolysis of VWF by ADAMTS13. Thus, RU8 may inhibit VWF proteolysis by inhibiting the initial binding of the distal domains of ADAMTS13 to the C-terminal region of VWF. We found that the mAb, SZ34, bound to native VWF better than pre-denatured VWF at the central A2 domain, and decreased the cleavage of native VWF under shear stress, but not pre-denatured VWF under static conditions. These findings suggest that SZ34 binds native VWF-A2 domain, which may block the conformational change of VWF or block the access of ADAMTS13 to the ancillary binding site and cleavage bond located at the central A2 domain under fluid shear stress.

The ADAMTS13 cleavage site (the Y1605-M1606 bond) in the VWF A2 domain is buried within the native, folded structure of VWF multimers and is not accessible to cleavage by the metalloprotease [3]–[5]. Although a report found that recombinant VWF-A2 peptide (a.a.1481–1668) was sensitive to proteolysis by ADAMTS-13 under physiological pH and non-denaturing conditions [17], most of studies have shown that even for the isolated A2 domain, unfolding by high fluid shear stress or chemical denaturants such as urea and guanidine-HCl is required for proteolysis by ADAMTS13 [10], [18]. Thus, for VWF with its multi-domain structure, not only the inter-domain but also the intra-domain A2 structural changes have regulatory roles in ADAMTS13-mediated cleavage of VWF. Our data demonstrate that the epitope of SZ34, a mAb raised against native VWF multimers, is located within the A1555-G1595 region, which comprises the α2-helix and α3-helix according to the crystal structure of VWF A2 domain [19]. It is possible that part or all of the α2-helix and α3-helix structure of A2 domain is constitutively exposed on the native VWF multimers and is involved in the regulation of VWF proteolysis by ADAMTS13. Previous studies, however, considered that the A2 domain of VWF is normally sandwiched between the much larger A1 and A3 domains [19], [20], and the D1596-R1668 region in VWF A2 domain (the so called VWF73) is the minimal substrate for ADAMTS13 [21].

We conclude that SZ34 is a conformationally sensitive anti-VWF mAb which can modulate proteolytic cleavage of VWF by ADAMTS13 under physiologically relevant conditions. This monoclonal anti-VWF antibody may be a useful tool for further investigating biological function of VWF *in vivo*.

## Materials and Methods

### Purification of plasma-derived VWF

Plasma-derived human VWF (pVWF) was purified from commercial VWF/FVIII concentrate by gel filtration with a Sepharose 4B-CL column (Amersham Pharmacia Biotech AB, Uppsala, Sweden) and heparin-Sepharose 6FF (Pharmacia) affinity chromatography, as reported [22]. VWF antigen (VWF:Ag) concentration was determined by an enzyme-linked immunosorbent assay (ELISA) kit from Dako (Glostrup, Denmark) and VWF multimers analysis was performed as reported [23].

### Recombinant human ADAMTS13

Plasmid ADAMTS13 containing human full-length cDNA sequence was generously provided by Dr. Jingfei Dong (Baylor College of Medicine, Houston, TX, USA) [24]. Recombinant ADAMTS13 (rADAMTS13) with the C-terminal His-tag was expressed in a stably transfected HeLa cell line. Expression medium was concentrated and purified using a Ni-NTA agarose column (QIAGEN GmbH, Hilden, Germany).

### Antibodies to VWF

Eight murine mAbs against human VWF were used. The anti-VWF D'D3 domain mAbs 1C1E7 and 75H4B12 were kind gifts from Dr. Deckmyn (Laboratory for Thrombosis Research, KU Leuven Campus Kortrijk, Kortrijk, Belgium) [25]. Others were all previously produced in our laboratory. SZ129 and SZ130 recognize VWF A1 domain [26], whereas SZ123 and SZ125 interact with VWF A3 domain [27]. SZ29 and SZ34 are mAbs to VWF, whose epitopes are indefinite because of being directly raised against purified plasma-derived full-length human native VWF [27], [28]. Horse-radish peroxidase (HRP)-conjugated polyclonal rabbit anti-human VWF IgG was purchased from Dako (Glostrup, Denmark).

### Cleavage of VWF by ADAMTS13 in the presence of anti-VWF mAbs under shear stress

Purified pVWF (150 nM) in 20 mM Tris-HCl (pH 8.0), 0.15 M NaCl, and 5 mg/ml (w/v) BSA was incubated separately with 8 anti-VWF mAbs (0–200 µg/ml) at 37°C for 30 min and then at 25°C for 3 min with 50 nM rADAMTS13 that had been activated by incubation with 5 mM CaCl_2_ in 20 mM Tris-HCl (pH 8.0), 0.15 M NaCl, and 5 mg/ml (w/v) BSA at 37°C for 1 h. The mixture was then subjected to a mini vortexer (Fisher Scientific, USA) with constant vortexing at 2,500 rpm for 5 min [23] and the reaction was terminated by the addition of 20 mM EDTA.

### Cleavage of VWF by ADAMTS13 in the presence of anti-VWF mAbs under static/denaturing conditions

Purified VWF (1.5 µM) was pre-denatured with 1.5 M guanidine-HCl in 20 mM Tris-HCl (pH 8.0) at 37°C for 2 h. After a 1∶10 dilution with 20 mM Tris-HCl (pH 8.0), the denatured VWF was incubated with anti-VWF mAbs (0–200 µg/ml) at 37°C for 30 min and then treated with 25 nM rADAMTS13 that had been activated by incubation with 5 mM CaCl_2_ at 37°C for 1 h. After incubation at 37°C for 1.5 h, the reaction was stopped by the addition of 20 mM EDTA.

### Expression of VWF-R1597W mutant and its cleavage by ADAMTS13 in the presence of mAb SZ34 under static/nondenaturing conditions

The plasmid *PSVHvWF1*, which harbors a full-length cDNA insert of human VWF (kindly provided by JE Sadler, Washington University School of Medicine, St Louis, USA) [29], was used to construct the R1597W mutant by PCR-based single-nucleotide mutagenesis. Recombinant VWF-R1597W mutant was expressed in transiently transfected Hela cell line using serum-free OPTI-MEM I (Invitrogen) for 48 h. The media were collected and added into EDTA-free 1× proteinase inhibitor Cocktail (Roche), followed by concentration using Amicon Ultra-15 Centrifugal Filter (Millipore). VWF antigen (VWF:Ag) concentration was determined by an ELISA kit (Dako).

Recombinant VWF-R1597W (150 nM) was incubated with 0 or 100 µg/ml SZ34 at 37°C for 30 min and then for 18 h with 25 nM rADAMTS13 at 37°C.

### Assessment of VWF proteolysis by SDS-PAGE and agarose gel electrophoresis

The cleavage reactions were measured by the distribution of proteolytic fragments (350 kDa) on a 5% SDS-PAGE under non-reducing condition or by VWF multimeric size distribution on a 1.5% agarose gel electrophoresis, and then analyzed by Western blotting with HRP-conjugated anti-VWF IgG (Dako) and visualized by chemiluminescence as reported [23], [30]. The intensity of the bands was analyzed using Image J software.

### Preparation of recombinant human VWF fragments

VWF D'D3 (S764P1247-H) expressed in BHK cells was a gift from Dr. Deckmyn (Laboratory for Thrombosis Research, KU Leuven Campus Kortrijk, Kortrijk, Belgium). The other recombinant VWF fragments including A1A2A3 (Q1238G1874-H), A1 (H-E1260P1467), A2 (H-G1481R1668), A3 (S1681R1877-H), A2-12 (GST-D1459D1596-H), A2-23 (VWF114, GST-A1555R1668-H), A2-1 (GST-D1459E1554-H), A2-2 (GST-A1555G1595-H) and A2-3 (VWF73, GST-D1596R1668-H) were all expressed in bacteria. Plasmids encoding different VWF fragments were all generated from pSVHVWF1 [29].

Recombinant VWF D'D3, A1A2A3, A1, A2 and A3 all contained a 6×His tag (NOVAGEN, San Diego, CA, USA) (Figure 5A). The preparation of VWF A1, A2 and A3 was described previously [26], [27], [31]. Plasmid construction, expression and purification of VWF A1A2A3 were similar to VWF A3 [27]. Two primers were used for amplifying VWF A1A2A3: 5′-gga tcc GCA GGA GCC GGG AGG C-3′ and 5′-ctc gag TCC AGA GCA CAG TTT GTG-3′ (Lowercase letters for *Bam*HI and *Xho*I sites). Four recombinant VWF domains including A1A2A3, A1, A2 and A3 were all expressed in inclusion bodies. After purification, protein refolding was achieved using an 8 to 0 M urea linear gradient.

Plasmids encoding A2-12, A2-23 (VWF114), A2-1, A2-2 and A2-3 (VWF73) (Figure 5B) were constructed similarly in the GST fusion vector pGEX-6P-1 (Amersham Biosciences, Piscataway, NJ). These recombinant plasmids were prepared by use of primers as follows: A2-12 (5′-cgg gat cc GAC CTT GCC CCT GAA GCC CCT C-3′ and 5′-cgg aat tc TCA GTG ATG GTG ATG GTG ATG ACC CTG GCT GAC CAA GAA GCT G-3′), A2-23 (5′-cgg gat cc GCA CAG TCC AAA GGG GAC ATC C-3′ and 5′-cgg aat tc TCA GTG ATG GTG ATG GTG ATG CCT CTG CAG CAC CAG GTC AGG A-3′), A2-1 (5′-cgg gat cc GAC CTT GCC CCT GAA GCC CCT C-3′ and 5′-cgg aat tc TCA GTG ATG GTG ATG GTG ATG CTC GCT GAA GGG GTA CTC CAC AG-3′), A2-2 (5′-cgg gat cc GCA CAG TCC AAA GGG GAC ATC C-3′ and 5′-cgg aat tc TCA GTG ATG GTG ATG GTG ATG ACC CTG GCT GAC CAA GAA GCT G-3′) and A2-3 (5′-cgg gat cc GAC CGG GAG CAG GCG CCC AAC C-3′ and ′-cgg aat tc TCA GTG ATG GTG ATG GTG ATG CCT CTG CAG CAC CAG GTC AGG A-3′). Lowercase letters indicate added restriction enzyme sites (*Bam*HI and *Hind*III) and the underlined sequence was the inserted C-terminal 6×His-tag. The GST fusion proteins were expressed and purified by chromatography on Ni-NTA agarose (QIAGEN GmbH, Hilden, Germany) as described [21]. All five recombinant GST fusion proteins were mainly expressed in soluble fractions at low temperatures.

### Unfolding of full-length VWF and VWF fragments for epitope mapping of mAb SZ34

Heat treatment and denaturization treatment with guanidine-HCl were used to unfold recombinant VWF fragments and pVWF. Purified pVWF was denatured by heating at 80°C for 20 min in a thermo-block heater (ThermoStat Plus, Eppendorf) as reported [32].

Recombinant VWF fragments and pVWF were denatured with guanidine-HCl [30] as following. Purified pVWF or various VWF fragments (10 µM) were incubated with 1.5 M guanidine-HCl at 37°C for 2 h. Then, guanidine-HCl treated VWF fragments and pVWF were diluted serially with 20 mM Tris-HCl (pH 8.0), 0.15 M NaCl. The binding affinity of denatured VWF and SZ34 was determined by an ELISA. Because the initial concentrations of pVWF and VWF fragments were high, the final concentration of guanidine-HCl in solution was <20 mM, which did not interfere with the binding activity of SZ34 (data not shown), consistent with what was reported by Tsai [30] in which he showed that guanidine-HCl at 24 mM did not interfere with the platelet aggregation.

### Affinity measurements of SZ34 with full-length VWF and various VWF fragments using ELISA

The native or denatured VWF fragments and pVWF at various concentrations were first incubated in solution with the antibody (SZ34 or SZ29) at constant concentration until equilibrium was reached, respectively. The denatured VWF fragments and pVWF were obtained by denaturization treatment with guanidine-HCl as above. The concentration of free antibody was then determined by an ELISA. The equilibrium dissociation constants (Kd) of antigen/antibody complexes in solution were obtained according to the equation [33]:
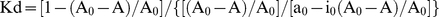
where A_0_ and A are the absorbances measured for the antibody in the absence and presence of antigen, respectively, and a_0_ and i_0_ respectively the total concentrations of antigen and antibody.

Increasing concentrations (0.5–100 nM) of the VWF fragments or pVWF with or without being denatured were mixed with a constant amount of SZ34 (0.3 nM), in 1% (w/v) BSA-PBS, respectively, until equilibrium were reached (15 h incubation at 22°C). Then the proportion of SZ34 which remained unsaturated at each concentration of various VWF fragments and pVWF was measured by an ELISA method as following. The antigen/antibody complexes were added to the microtiter plates coated with 100 µl of pVWF (7.5 µg/ml) and incubated at 37°C for 2 h. After being washed, the wells were incubated at 37°C for 2 h with HRP-labeled goat anti-mouse IgG (Sigma) in 1% (w/v) BSA-PBS. The plates were washed 5 times and tetramethyl benzidine (TMB) solution was added for color development. Absorbance was read at 450 nm and A_0_ and A were then obtained, respectively.

### Binding of SZ34 to native and unfolded VWF by Western blot/ELISA based on polystyrene microspheres

To distinguish the linear from the conformational epitope of SZ34, we used an in-house Western blot/ELISA (Western blot in combination with ELISA) based on polystyrene microspheres. SZ129 (a mAb to VWF A1 domain) was used as a control. Briefly, 20 µg/ml of SZ34 or SZ129 in 0.05 M sodium carbonate buffer (pH 9.6) was mixed with 1∶10 volume of 50 mg/ml polystyrene microsphere suspension (SDL37; Takeda Chemical Industries, Co., Ltd., Osaka, Japan). The mixtures were incubated at room temperature for 2 h with shaking. The microspheres were then centrifuged and resuspended in a two-fold dilution of the starting volume of 2.5% (w/v) BSA-PBS to block the free binding sites. This solution was incubated at room temperature for 2 h with constant shaking. After washing three times with PBS, the microspheres were incubated with 100 nM of pVWF with or without denaturing at room temperature for 1 h with gentle agitation. The microspheres were washed three times with PBS, added in sample buffer (0.0625 M Tris-HCl buffer, pH 6.8, 10% (v/v) glycerol, 2% (w/v) SDS, 5% (v/v) β-mercaptoethanol, 0.001% (w/v) bromophenol blue, 4 mM EDTA), and then heated at 99°C for 5 min. After centrifugation at 10,000 g for 3 min, the supernatants were subjected to 6% SDS-PAGE. The proteins were transferred to membranes and blotted with HRP-conjugated anti-VWF IgG (Dako).
