# Correction: A Conformation-Sensitive Monoclonal Antibody against the A2 Domain of von Willebrand Factor Reduces Its Proteolysis by ADAMTS13

**DOI:** 10.1371/annotation/fe2ceb97-9332-4479-9827-90dcd4027570

**Published:** 2011-09-23

**Authors:** Jingyu Zhang, Zhenni Ma, Ningzheng Dong, Fang Liu, Jian Su, Yiming Zhao, Fei Shen, Anyou Wang, Changgeng Ruan

The authors would like to add the following to their financial disclosure: "The study is also a Project Funded by the Priority Academic Program Development of Jiangsu Higher Education Institutions." 

